# Cultivar-Dependent Anticancer and Antibacterial Properties of Silver Nanoparticles Synthesized Using Leaves of Different *Olea Europaea* Trees

**DOI:** 10.3390/nano9111544

**Published:** 2019-10-30

**Authors:** Valeria De Matteis, Loris Rizzello, Chiara Ingrosso, Eva Liatsi-Douvitsa, Maria Luisa De Giorgi, Giovanni De Matteis, Rosaria Rinaldi

**Affiliations:** 1 Department of Mathematics and Physics “Ennio De Giorgi”, University of Salento, Via Arnesano, 73100 Lecce, Italy; marialuisa.degiorgi@unisalento.it (M.L.D.G.); dematteis.giovanni@virgilio.it (G.D.M.); ross.rinaldi@unisalento.it (R.R.); 2 Department of Chemistry, University College London, 20 Gordon Street, London WC1H 0AJ, UK; lrizzello@ibecbarcelona.eu (L.R.); eva.liatsi-douvitsa.14@ucl.ac.uk (E.L.-D.); 3 Institute for Bioengineering of Catalonia (IBEC), The Barcelona Institute of Science and Technology, Baldiri Reixac 10-12, 08028 Barcelona, Spain; 4 CNR-IPCF S.S. Bari, c/o Department of Chemistry, Università degli Studi di Bari, via Orabona 4, I-70126 Bari, Italy; c.ingrosso@ba.ipcf.cnr.it

**Keywords:** Green synthesis, Silver nanoparticles, *Olea Europaea*, *Leccino*, *Carolea*, cytotoxicity, genotoxicity, antibacterial activity

## Abstract

The green synthesis of nanoparticles (NPs) is currently under worldwide investigation as an eco-friendly alternative to traditional routes (NPs): the absence of toxic solvents and catalysts make it suitable in the design of promising nanomaterials for nanomedicine applications. In this work, we used the extracts collected from leaves of two cultivars (*Leccino* and *Carolea*) belonging to the species *Olea Europaea*, to synthesize silver NPs (AgNPs) in different pH conditions and low temperature. NPs underwent full morphological characterization with the aim to define a suitable protocol to obtain a monodispersed population of AgNPs. Afterwards, to validate the reproducibility of the mentioned synthetic procedure, we moved on to another Mediterranean plant, the *Laurus Nobilis*. Interestingly, the NPs obtained using the two olive cultivars produced NPs with different shape and size, strictly depending on the cultivar selected and pH. Furthermore, the potential ability to inhibit the growth of two woman cancer cells (breast adenocarcinoma cells, MCF-7 and human cervical epithelioid carcinoma, HeLa) were assessed for these AgNPs, as well as their capability to mitigate the bacteria concentration in samples of contaminated well water. Our results showed that toxicity was stronger when MCF-7 and Hela cells were exposed to AgNPs derived from *Carolea* obtained at pH 7 presenting irregular shape; on the other hand, greater antibacterial effect was revealed using AgNPs obtained at pH 8 (smaller and monodispersed) on well water, enriched with bacteria and coliforms.

## 1. Introduction

The growing use of nanotechnology-based materials is providing new solutions to previously unsolved issues [1]. AgNPs represent the 24 % [2] of the whole materials present in the current textiles, plastics, food, and other countless commercial products [3,4,5,6]. Their use is even higher in electronics [7], medicine [8], and materials sciences [9]. These widespread applications are mainly due to their unique physico-chemical properties that ranged from plasmonic [10] to antibacterial [11] and anticancer activity [12]. Inevitably, their potential impact on human health as well as on the environment, especially in terms of dangerous chemicals used for producing any nanomaterial were investigated. AgNPs can be synthesized by means of physical or chemical methods [13]. The latter chemical approach is based on the use of either organic or water solutions containing metal precursors and reducing agents. The typical drawback here is the toxicity induced by the chemicals, that thus required the implementation of long and time-consuming post-synthesis purification procedures [14]. Despite chemical and physical routes represent the most common methods used to synthetize high quality AgNPs, finding new “green” solutions for their development will represent a serious step towards a decrease of environmental impacts [15]. Green chemistry is an eco-friendly and valid alternative due to the use of natural agents for the production of NPs [16]. This eco-friendly alternative employs natural products and non-toxic agents, which are usually in the form of plant extracts or even microorganisms and water [17]. Green synthesis routes show several pros, such as: (*i*) reducing the waste products, (*ii*) decreasing the energy-associated cost of productions and (*iii*) eliminating the environmental toxicity [18]. In many green syntheses, the silver ions (Ag^+^) reduction, that is the crucial step for the assembling of AgNPs, is mediated by the biomolecules presents in the plants extract (i.e., proteins, alkaloids, saponins, amino polysaccharides proteins, tannins, enzymes, vitamins etc)[19,20]. Another advantage consists in the Ag^+^ reduction in plant extracts-mediated syntheses, with a faster rate compared to the process performed with microorganisms [21]. Many works are available on the bio-synthesis of AgNPs from plant leaves extracts [22], such as *Rosa rugosa* [23], *Psidium guajava* [24], *Magnolia Kobus, Ginko biloba*, *Pinus desiflora*, *Diopyros kaki*, *Pllatanus orientalis* [25], *Stevia rebaudiana* [26], *Cocos nucifera coir* [27], *Chenopodium album* [28], *Gliricidia sepium* [29], *Ocimum sanctum* [30], *Cycas* [31]. *Olea Europaea* is one of the most important tree in the Mediterranean countries, due to their capability to produce oil, a relevant source of nutrition and medicine [32]; the leaves have always been used as antimalarial, antibacterial and anti-mycoplasma agent, thanks to their inherently high concentration of antioxidants and anti-inflammatory molecules [33]. Biological molecules such as luteolin-7-glucoside, luteolin, cafeic acid, p-coumaric acid, vanillin, diosmetin, rutin, apigenin-7-glucoside, diosmetin-7-glucoside and vanillic acid [34,35] are present in high concentrations [36]. Secoiridoids like oleuropein only exist in plants belonging to the family of *Oleaceae*. These compounds are both terpenic and hydroxy aromatic secondary metabolites. Olive leaves are also rich in mannitol, which is a sugar alcohol, synthesised only by the *Oleaceae* plants [37]. Therefore, in this study, we wondered if two *Olea Europaea* cultivar leaves, *Leccino* and *Carolea*, might be used as natural sources to synthesize AgNPs with controlled properties. We used the leaves of these trees resulting from pruning and other agricultural activities, which were destined to become waste. We investigated the physico-chemical properties of the AgNPs achieved, from the two selected cultivar leaves, in low temperature conditions and different pH conditions (pH 7 and pH 8). Such a green synthetic route, optimized for achieving AgNPs from olive oil extracts, was found to be fast, low-cost, reproducible and environmentally friendly. In addition, its reproducibility was assessed by synthesizing AgNPs from another Mediterranean plant, the *Laurus Nobilis*. Finally, the antibacterial and anticancer properties of the obtained AgNPs, were tested against bacteria colonized well water and two cancer cell lines, respectively.

## 2. Materials and Methods

### 2.1. Preparation of Leaves Extract

Leaves of two *Olea Europaea* cultivar (*Leccino* and *Carolea*), were collected in winter from olive trees. Several washes with MilliQ water were carried out in order to remove pollution or other contaminants from leaves. Leaves were then dried at room temperature overnight. There was 50 g of leaves finely cut and added in 500 mL of MilliQ water. The mix was boiled for 15 min. The extract was cooled down to room temperature, and afterwards filtered with Whatman No. 1 filter paper. The filtrate was further centrifuged at 4000 rpm for 10 min and the supernatant was used for the synthesis. The same procedure was implemented for the preparation of *Laurus Nobilis* extract.

### 2.2. Synthesis of Green AgNPs

There was 5 mL of extract added to 100 mL of AgNO_3_ (1 mM) and the reaction was heated to 60 °C for 45 min. In this time, the reaction colour changed from clear yellow to dark brown, indicating reduction of Ag^+^ ions to Ag^0^ NPs at pH 7. We also used NaOH to increase the pH of the mixture from 7 to 8. The solution was centrifuged at 12.000 rpm for 30 min in order to obtain concentrated AgNPs for the next characterizations steps.

### 2.3. Characterization of Green AgNPs

#### 2.3.1. Transmission Electron Microscopy (TEM), Dynamic Light Scattering (DLS) and ζ-Potential

TEM characterizations were carried out with a JEOL Jem 1011 microscope, operating at an accelerating voltage of 100 kV (JEOL USA, Inc, Peabody, MA, USA). TEM samples were prepared by dropping a dilute solution of NPs in water on carbon-coated copper grids (Formvar/Carbon 300 Mesh Cu). DLS and ζ-potential measurements were performed on a Zetasizer Nano-ZS equipped with a 4.0 mW HeNe laser operating at 633 nm and an avalanche photodiode detector (Model ZEN3600, Malvern Instruments Ltd., Malvern, UK). Measurements were performed at 25 °C in aqueous solution (pH 7).

ImageJ open source software (NIH image) version 1.47 was used with a suite of analysis routines used for particle analysis to test the circularity values of NPs measured on TEM acquisition. Sorting based on circularity and including only those with circularity values > 0.8 will ensure any aggregates are not included in the measurement [38].

#### 2.3.2. UV-Vis Spectroscopy

The UV–Vis absorption spectra of AgNPs samples were collected at room temperature by means of a Cary 5000 UV/Vis/NIR spectrophotometer (Varian, Palo Alto, CA, USA).

#### 2.3.3. Energy-Dispersive X-ray Spectroscopy (EDS)

EDS analyses were recorded with a Phenom ProX microscope (Phenom-World B. V., Eindhoven, Germany), at an accelerating voltage of 10 kV. The samples were prepared by dropping a solution of NPs in water onto monocrystalline silicon wafer.

#### 2.3.4. Attenuated Total Reflection (ATR) Fourier Transform Infrared Spectroscopy (FTIR)

Mid-infrared spectra were acquired with a Varian 670-IR spectrometer equipped with a DTGS (deuterated tryglycine sulfate) detector. The spectral resolution used for all experiments was 4 cm^−1^. For attenuated total reflection (ATR) measurements, a one-bounce 2 mm diameter diamond microprism was used as the internal reflection element (IRE). Films were directly cast onto the internal reflection element by depositing the solution or suspension of interest onto the upper face of the diamond crystal and allowing the solvent to evaporate.

#### 2.3.5. Cell Culture

MCF-7 and Hela were maintained in high glucose DMEM with 50 μM of glutamine, supplemented with 10% FBS, 100 U/mL of penicillin and 100 mg/mL of streptomycin. Cells were incubated in a humidified controlled atmosphere with a 95 % to 5 % ratio of air/CO_2_, at 37 °C.

#### 2.3.6. WST-8 Assay

MCF-7 and Hela cells were seeded in 96 well microplates at the concentration of 5 × 10^3^ cells/well after 24 h of stabilization. NPs stock solutions (AgNPs from the *Leccino*, *Carolea* at pH 7 and AgNPs from the *Leccino*, *Carolea* and *Laurus Nobilis* at pH 8) were added to the cell media at 20 µg/mL and 50 µg/mL. Cells were incubated for 48 and 96 h. At the endpoint, cell viability was determined using a standard WST-8 assay (Sigma Aldrich). Assays were performed following the procedure previously described in De Matteis et al [39]. Data were expressed as mean ± SD.

#### 2.3.7. Lactate Dehydrogenase (LDH) Assay

MCF-7 and Hela cells were seeded in 96 well microplates (Constar) and treated with NPs stock solutions (AgNPs from the *Leccino*, *Carolea* at pH 7 and AgNPs from the *Leccino*, *Carolea* and *Laurus Nobilis* at pH 8) at 20 µg/mL and 50 µg/mL of concentration. After 48 and 96 h of cell–AgNP interaction, the LDH leakage assay was performed onto microplates by applying the CytoTox-ONE Homogeneous Membrane Integrity Assay reagent (Promega) following the manufacturer’s instructions. The culture medium was collected, and the level of LDH was measured by reading absorbance at 490 nm using a Bio-Rad microplate spectrophotometer (Biorad, Hercules, CA, USA). Data were expressed as mean  ±  SD.

#### 2.3.8. Determination of the Intracellular Uptake of Green AgNP_S_ by Inductively Coupled Plasma Atomic Emission Spectroscopy (ICP-AES)

1 × 10^5^ of MCF-7 and HeLa cells were seeded in 1 mL of medium in a 6-well plate. After 24 h of incubation at 37 °C, the medium was replaced with fresh medium containing the green AgNPs obtained at pH 7 and pH 8, at the concentrations of 20 µg/mL and 50 µg/mL. After 48 h and 96 h of incubation at 37 °C, the culture medium was removed, and the MCF-7 and Hela washed with PBS buffer to eliminate non-internalized NPs. Cells were detached with trypsin and counted by sing automatic cell counting chamber. 360.000 cells were suspended in 200 µL of milliQ, and treated with HNO_3_ and diluted to 5 mL: the solution was analysed to evaluate Ag content. Elemental analysis was carried out by ICP-AES, Varian Vista AX spectrometer (Varian Inc., Palo Alto, CA, USA).

#### 2.3.9. Comet Assay (Single Gel Electrophoresis)

HeLa cells were exposed to 50 µg/mL of AgNPs obtained from *Leccino* and *Carolea* for 96 h, at density of 5 × 10^4^ in each well of 12-well plates in a volume of 1.5 mL. After treatments, cells were centrifuged and suspended in 10 μL of PBS at concentration of 1000 cells/μL. The cell pellets were mixed with 75 μL of 0.75 % low-melting-point agarose (LMA) and then layered onto microscope slides pre-coated with 1% normal melting agarose (NMA) and dried at room temperature. Subsequently, the slides were immersed in an alkaline solution (300 mM of NaOH, 1 mM of Na_2_EDTA, pH 13) for 20 min to allow for unwinding of the DNA. The electrophoresis was carried out in the same buffer for 25 min at 25 V and 300 mA (0.73 V/cm). After electrophoresis, cellular DNA was neutralized by successive incubations in a neutralized solution (0.4 MTris–HCl, pH 7.5) for 5 min at room temperature. The slides were stained with 80 μL SYBR Green I (Invitrogen). Comets derived from single cells were photographed under a Nikon Eclipse Ti fluorescence microscope, and head intensity/tail length of each comet were quantified using Comet IV program (Perceptive Instruments).

#### 2.3.10. Determination of Ag^+^ Release

Ag^+^ release was quantified using 50 µg/mL of AgNPs from *Leccino* and *Carolea* cultivar obtained at pH 7 and AgNPs from *Leccino*, *Carolea* cultivar and *Laurus Nobilis* obtained at pH 8. The release was studied upon 24 h, 48 h and 96 h of incubation time in water (pH 7) and acidic buffer (pH 4.5). After the time points, the NPs were collected by centrifugation at 13.000 rpm for 1 h and digested by the addition of HNO_3_ solution (10% v/v). The number of free ions was measured by ICP-AES (Varian Inc., Palo Alto, CA, USA).

#### 2.3.11. Confocal Measurements

HeLa cells were seeded at concentration of 8 × 10^4^ cells/mL in glass Petri dishes (Sarstedt, Germany). After 24 h of stabilization, the culture media was supplemented with AgNPs derived from *Leccino* and *Carolea* (50 µg/mL) for 96 h. After exposure, the medium was removed; then three washes with Phosphate Buffered Saline (PBS, D1408, Sigma Aldrich) were performed. Samples were fixed by using glutaraldehyde (G5882, Sigma Aldrich) at 0.25 % in PBS for 10 min. After two washes with PBS, Triton X-100 (Sigma Aldrich) at 0.1 % for 5 min was used to permeabilize the cell membrane of fixed cells before staining the nuclei by 1 μg/mL of DAPI (D9542, Sigma Aldrich) for 5 min. Acquisitions were performed by Leica TCS SPE-II confocal microscope using a 100× objective (water immersion, HCX PL APO, 1.10NA). The fluorescent images were obtained exciting fluorescent dyes by means laser radiation having wavelength at 405 nm. The nuclear morphology was quantified in terms of shape descriptor parameter: circularity. Circularity parameter compares an object to a circle; it is ranges from 0 to 1 (for a perfect circle). All results were obtained as means calculated on 15 cells and data were statistically analysed by means of a paired two-tailed *t*-test. The statistical difference of results was considered significant for *p*-value < 0.05*.

### 2.4. Antibacterial Activity of Green AgNPs

#### Collection of Water Samples

Water samples were collected from coliforms contaminated artesian well in south of Italy with bottles previously sterilized. The collection was done in the early morning, because it was reported that the coliforms could increase in warm pulled water [40].

### 2.5. Total Bacteria Detection by Plate Count Techniques

The count of viable bacteria was performed by plate count techniques [41].Well water (1 mL) was dropped on petri dishes previously filled with 9 mL of Plate Count Agar (Liofilchem) using spread plate technique for control. For treated samples, 50 µg/mL of green AgNPs derived both from *Leccino* and *Carolea* at pH 7 and from *Leccino*, *Carolea* and *Laurus Nobilis* at pH 8 were added. The inoculated plates were incubated at 22 °C for 72 h and 37 °C for 48 h after which the plates were observed. Bacteria growth and numbers of colonies were counted using a colony counter. Colony counts were expressed as Colony Forming Units (CFU/ml) of the sample:

No. of CFU/ml = No. of colonies counted × Dilution factor × Volume of sample taken.

### 2.6. Most Probable Number (MPN) to detect Coliforms and Faecal Coliforms

Most Probable Number (MPN) method [42] permitted to evaluate the number of coliforms bacteria in well water by means of replicate liquid broth growth in ten-fold dilutions. Contaminated well water was diluted serially and inoculated in Lactose Broth (Merck) at 37 °C for 24 h. The treated well water samples were represented by water with 50 µg/mL of green AgNPs derived both from *Leccino* and *Carolea* at pH 7 and from *Leccino*, *Carolea* and *Laurus Nobilis* at pH 8 before the inoculation at 37 °C for 24 h. The presence of total coliforms in water was detected by the ability of these bacteria to produce acid and gas using lactose. The acid production was detected by color change and the gas with the gas bubbles in the inverted Durham tube. To evaluate the AgNPs-induced bacteria reduction, the total number of coliforms, in terms of MPN index (estimated number of coliforms in 100 mL of water), was obtained by counting the tubes within two reactions taking place and comparing them with standard statistical tubes. This involved the presumptive, confirmed and completed test for coliform bacteria [43]. Incubation at high temperature was used to distinguish organisms of the total coliforms from faecal coliform group. In order to detect faecal coliforms, 1 mL of liquid medium from the tubes that underwent a color change, indicating the presence of coliforms, was added to EC broth (10 mL) at 44.5 °C for 24 h. Gas production with growth within 24 ± 2 h of incubation at 44.5 ± 0.2 °C is considered positive for the presence of faecal coliforms in water. Absence of gas production is considered a negative test for the presence of faecal coliforms [44,45].

## 3. Results and Discussion

**Figure 1:**
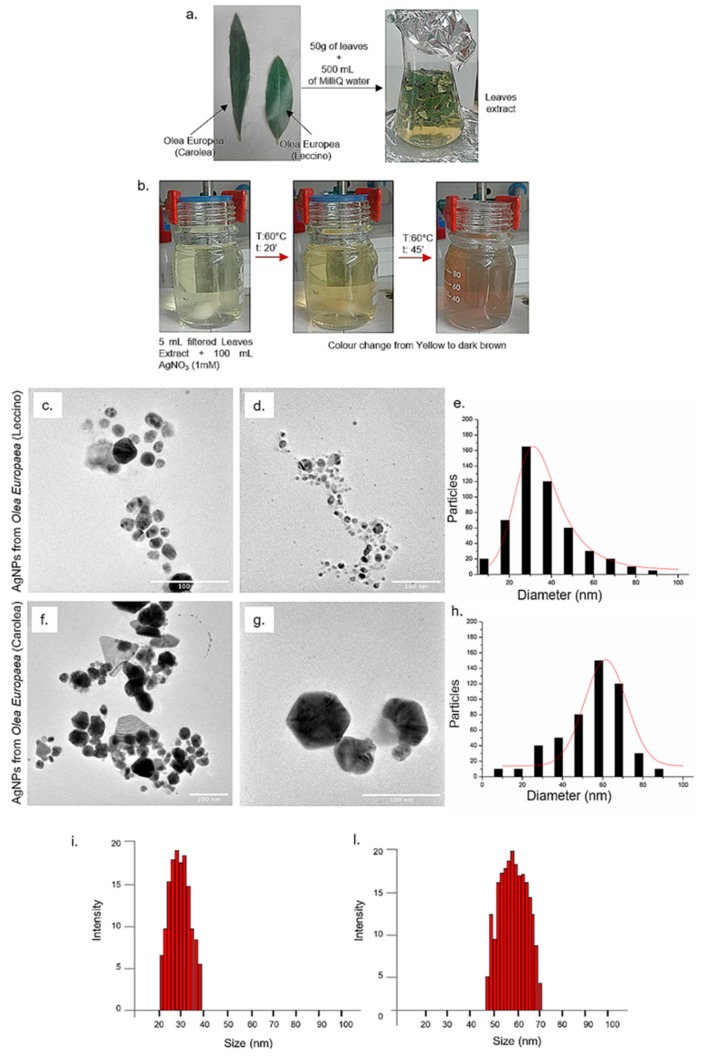
Morphologic differences between leaves collected from *Leccino* and *Carolea* cultivars and leaves extract preparation (**a**). The reaction started with AgNO_3_ addition and the change of colour from yellow to dark brown indicated silver NPs (AgNPs) formation (**b**). Representative TEM images of AgNPs obtained from *Olea Europaea Leccino* (**c**–d) and from *Carolea* (**f**–**g**) at pH 7. Size distribution was measured on 500 AgNPs from *Leccino* (**e**) and *Carolea* (**h**) and fitted with a normal function (solid line). Dynamic Light Scattering (DLS) measurements in water of AgNPs from *Leccino* (**i**) and *Carolea* (**l**) at pH 7.

The use of plants or microorganisms to synthetize NPs is a method particularly suitable for achieving metal NPs, such as AgNPs [46]. In our work, we used plants extracts obtained from leaves of typical Mediterranean tree, which can be easily found in Italy, namely *Olea Europaea*. The olive is an evergreen tree and its leaves are by products of olive farming that are stored during the pruning process [47]. Among different cultivar, differences in leaves length can be observed ranging from 30 to 80 mm [48]. The leaves from *Olea Europaea*, *Leccino* and *Carolea* cultivar, growing in the same pedoclimatic conditions (Figure 1a), were collected in winter when the bioactive compounds, such as amino acids, tannins and carbohydrates content was abundant [49]. In particular, the production of NPs was favoured by the high concentration of phenols in cold season that helped the Ag^+^ clustering, which was the seeding event in the growth of NPs [50]. Leaves were used to prepare plants extracts (Figure 1a) useful to obtain AgNPs with easy and not-toxic reproducible synthetic route with the addition of 1 mM of AgNO_3_ at two different pH (7 and 8) and at low temperature. The solution turned dark brown in 45 min confirming the AgNPs formation promoted by reduction of the Ag^+^ (Figure 1b). The dark brown colour indicated the free conduction electrons oscillation induced by the surface plasmon resonance excitation phenomenon [51,52].

**Figure 2:**
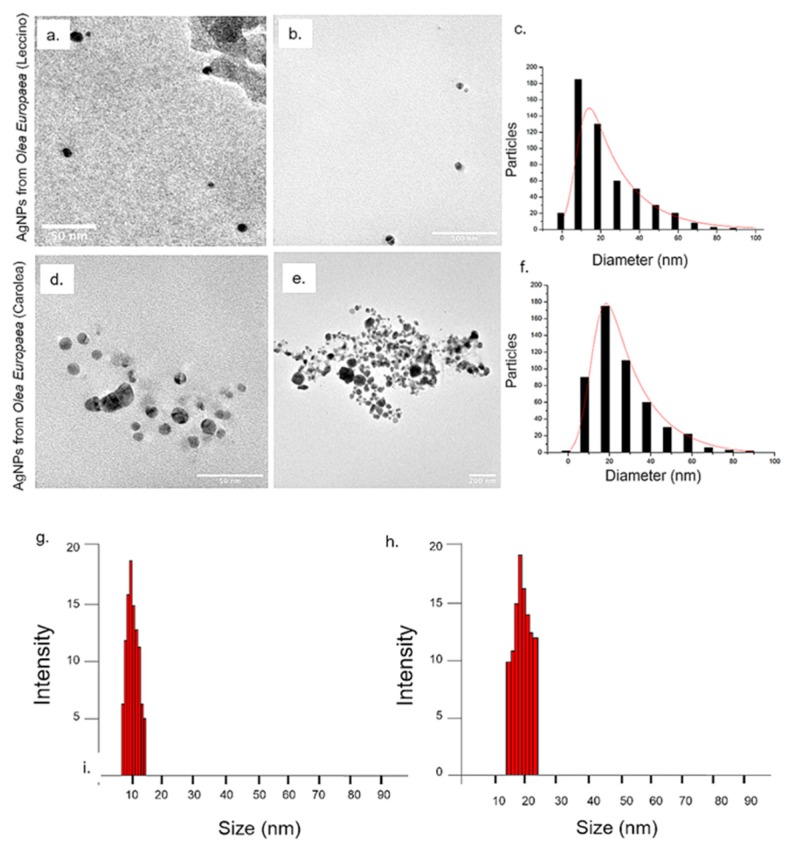
Representative TEM images of AgNPs obtained from *Olea Europaea* (**a**–**b**) *Leccino* and (**d**–**e**) *Carolea* at pH 8. Size distribution was measured on 500 AgNPs from *Leccino* (**c**) and *Carolea* (**f**) and fitted with a normal function (solid line). DLS measurements in water of AgNPs from *Leccino* (**g**) and *Carolea* (**h**) at pH 8.

The difference between the newly suggested green synthetic route and the previous approach by some of the authors [39] consists in the non-use of in sodium citrate and tannic acid, added at high temperature, to boost the Ag^+^ reduction to obtain stable and monodispersed spherical AgNPs with a size of (20 ± 3) nm. NPs obtained from leaves extracts were deeply characterized by means of TEM, DLS, ζ-Potential, UV-vis, EDS and FTIR-ATR in water. TEM analyses confirmed that AgNPs were different in shape and size when using the two cultivar extracts at pH 7 (Figure 1c,d,f,g). In detail, AgNPs derived from *Leccino* at pH 7 showed a quasi-spherical morphology and a mean size of (35 ± 8) nm (Figure 1e), whereas AgNPs obtained from *Carolea* were mainly triangular and hexagonal in shape, with a mean size of (60 ± 11) nm (Figure 1h). DLS measurements carried out in water were perfectly consistent with TEM analyses: in fact, the AgNPs showed a hydrodynamic radius compatible with the mean size values noticed in TEM acquisitions. In particular, at pH 7, the hydrodynamic radius recorded for *Leccino* was (31 ± 9) nm (Figure 1i) whereas it was (58 ± 14) nm for *Carolea* (Figure 1l). Interestingly, the increase of the pH reaction solution up to 8 triggered the formation of monodispersed AgNPs with comparable size and shape, though using different *Olea Europaea* cultivars; in fact, spherical shape and smaller mean size (10 - 22 nm) were observed in NPs synthesized using extracts from both cultivars. Such figures of merit were indeed different compared to those observed in NPs synthesized at pH 7 (Figure 2a,b,d,e). These results were correlated to pH 8 that influenced the stabilization NPs [22]. AgNPs from *Leccino* had a mean size of (15 ± 2) nm (Figure 2c). On the contrary, the same NPs derived from *Carolea* showed a mean size of (23 ± 7) nm (Figure 2f). DLS measurements allowed an estimation of the NP hydrodynamic radius of (12 ± 3) nm for NPs from *Leccino* (Figure 2g), and 20 ± 8 nm from *Carolea* (Figure 2h). Such values were in agreement with the TEM observations.

**Figure 3:**
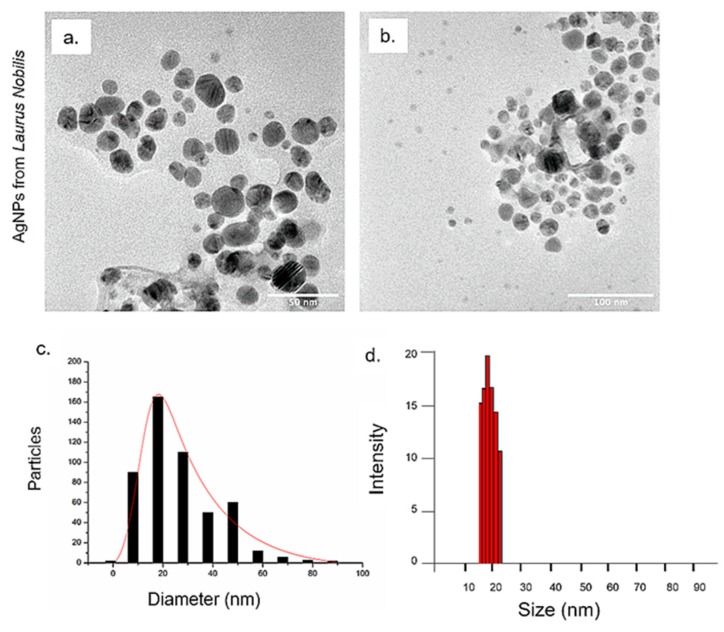
Representative TEM images of AgNPs obtained from *Laurus Nobilis* at pH 8 (**a**–**b**). Size distribution was measured on 500 AgNPs and fitted with a normal function (solid line) (**c**). DLS measurements in water of AgNPs from *Laurus Nobilis* at pH 8 (**d**).

After demonstrating the potential to use leaves from *Olea Europaea* for the synthesis of AgNPs, we moved on to synthesize AgNPs from the extract of *Laurus nobilis,* in order to validate the results obtained by using the same procedure at pH 8 using extracts from different trees deriving from the same Mediterranean area. In this case, the achieved AgNPs showed a mean size of (20 ± 8) nm (Figure 3c), as also confirmed by the DLS peak at (22 ± 6) nm (Figure 3d). These data were consistent with those found for the above reported syntheses at the same pH, using olive leaves extracts.

Afterwards, ζ-potential analyses revealed that negative surface charge was observed for all the obtained AgNPs in water (Table 1). This can be ascribed to proteins and other biological molecules present in leaves extracts, adsorbed on the NPs surface [53]. In fact, during green synthetic process, biomolecules such as proteins and peptides behave as capping agents and they are typically adsorbed during the NPs formation step, affecting the reaction dynamic and the NPs growth in different directions. These negatively charged natural capping agents are responsible of NPs stabilization due to their ability to control particles size, shape/morphology and to protect the surface from agglomeration phenomena that will influence their consequent uptake in cells [54].

Image J software was used to investigate the NPs sharpness by means the circularity parameter. We used high resolution TEM images to measure the geometrical parameters of the NPs; 50 random NPs from each type were investigated to obtain the average circularity distribution (Table 2). Circularity values near 1 indicate a perfect cycle, whereas near 0 an high sharpness degree [55]. The AgNPs obtained from *Carolea* cultivar at pH 7 had an average circularity of (0.28 ± 8) and showed a much higher degree of sharpness when compared to AgNPs from *Leccino* at the same pH 7, which presented an average circularity of (0.55 ± 4). NPs tended to assume a more spherical morphology at pH 8 even if different cultivars were used in basic synthesis, with an average circularity of (0.88 ± 3) for *Leccino*, and (0.63 ± 6) for *Carolea*. *Laurus nobilis* extract, on the other hand, induced the formation of AgNPs with a circularity value of (0.65 ± 4).

**Figure 4:**
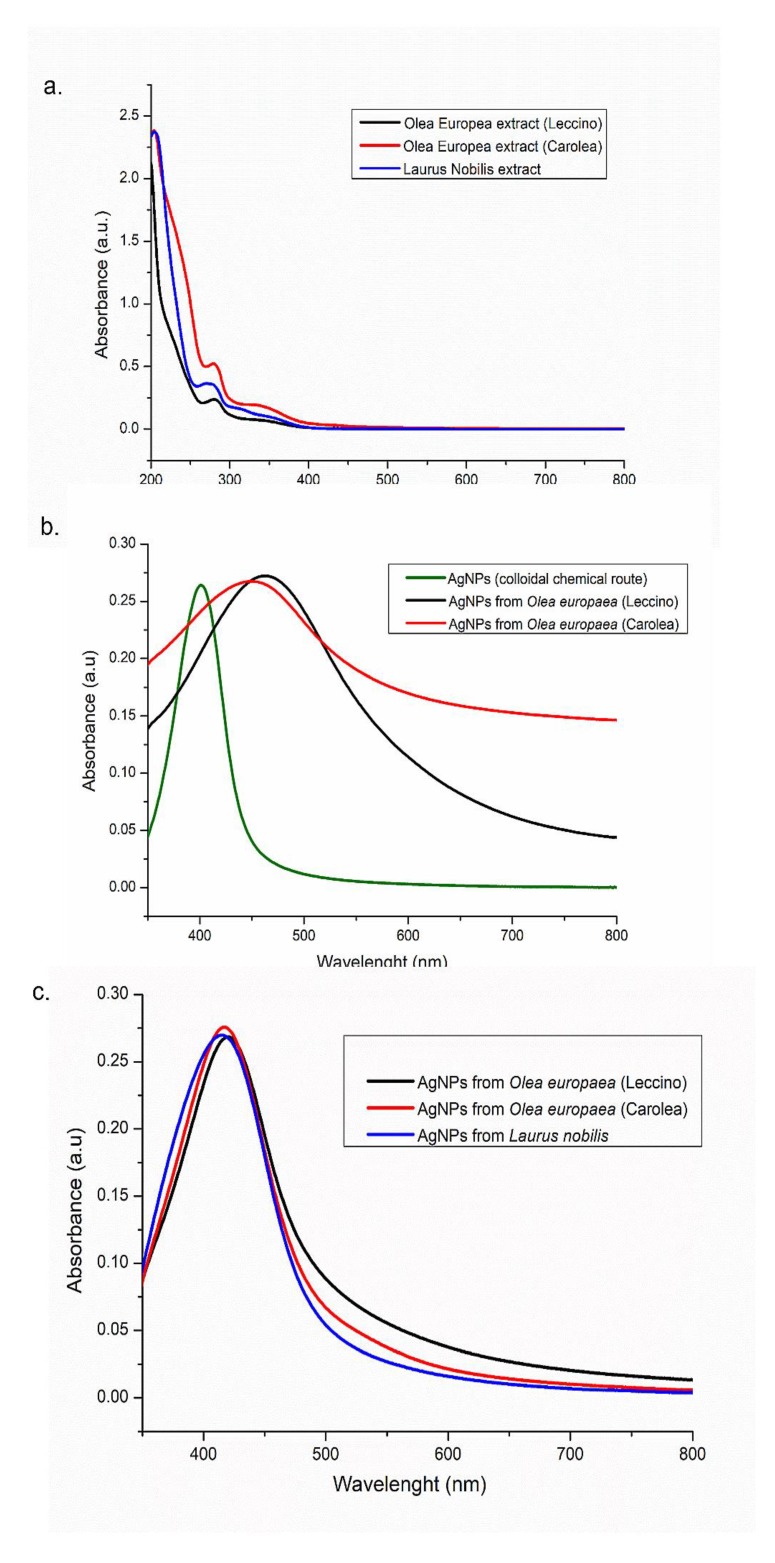
UV-vis spectra of *Olea Europaea* extracts (*Leccino*, *Carolea*) and *Laurus Nobilis* (**a**), UV-vis spactra of AgNPs derived from colloidal chemical routes and from green synthesis using *Olea Europaea* extracts (*Leccino* and *Carolea*) at pH 7 (**b**), AgNPs derived from green synthesis using *Olea Europ*aea extracts (*Leccino* and *Carolea*) and *Laurus Nobilis* at pH 8 (**c**).

UV-vis absorption spectra of the AgNPs were recorded in the 300–800 nm range and compared with those of the corresponding leaves extracts (Figure 4a).

1 mg/mL of leaves extracts, prepared as reported in the experimental section was used, and two peaks in the UV region, namely at 280 nm and 350 nm, probably due to aromatic compounds, have been detected for all the extracts solutions, while no absorption signal was detected in the 400-800 nm range. The absorption spectra of the AgNPs obtained from *Leccino* and *Carolea* extracts at pH 7 showed a surface plasmon resonance peak at 463 nm and 458 nm, respectively, which was red-shifted compared to AgNPs synthesized by colloidal chemical routes [39], showing a peak at 400 nm. It is worthwhile to notice that in both samples; the plasmon peaks were rather broad, suggesting the formation of AgNPs with a broad size distribution (Figure 4b). In particular, when the sharpness degree increased like in the case of triangulary-shaped NPs, the spectrum underwent a pronounced red shifted with respect to the AgNPs produced by colloidal chemical reduction processes. When the synthesis was performed at pH 8, the absorption spectra were narrow and closer to the wavelength absorption peak of the AgNPs synthesized by colloidal chemical routes. Namely, a peak at 420 nm was observed for the AgNPs from *Leccino*, and at 417 nm for the AgNPs from *Carolea*. The absorption peak of the AgNPs from *Laurus nobilis* was at 415 nm (Figure 4c).

**Figure 5:**
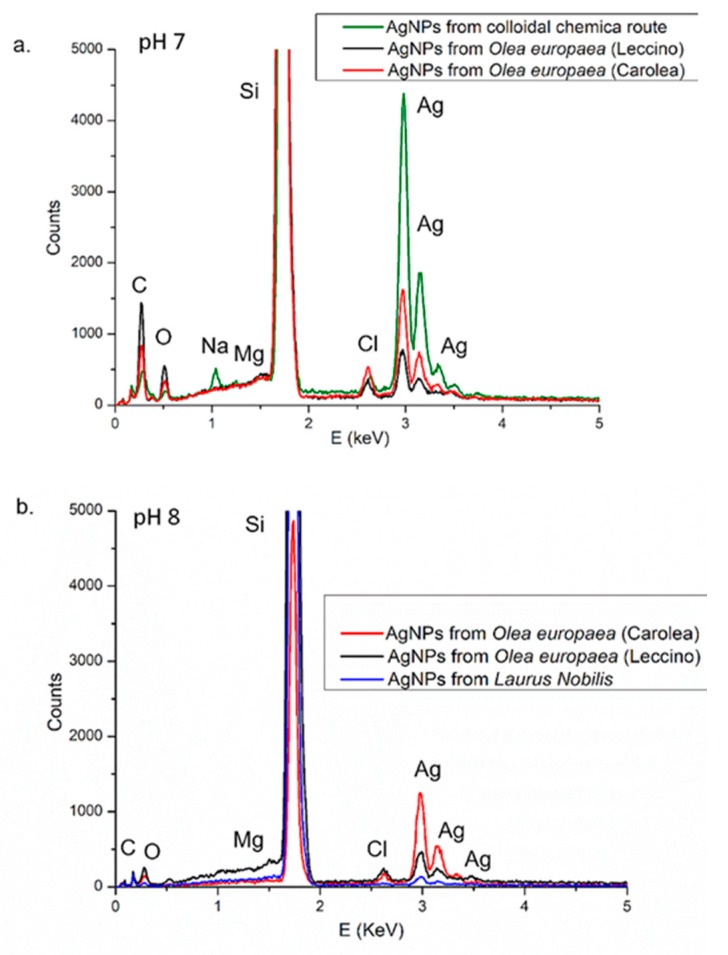
EDS measurements of AgNPs obtained from colloidal chemical route, from *Leccino* cultivar and *Carolea* at pH 7 (**a**) and from *Leccino* cultivar, *Carolea* cultivar and *Laurus Nobilis* at pH 8 (**b**).

Elemental analyses were also performed to investigate the chemical composition of the NPs samples. The EDS analyses of the AgNPs deposited onto silicon substrate in the range of 0–5 keV (Figure 5) clearly showed a strong spectral signal in the silver region (3–3.5 keV), both for NPs derived from colloidal chemical route and from green NPs, processed both at pH 7 and pH 8. The signals related to Na, Mg, Cl, C, O suggested the presence of biomolecules (carbohydrates and proteins). The Na element can be originated from sodium citrate, which was used for the colloidal chemical synthesis (green spectrum in Figure 5a).

FTIR-ATR spectroscopy investigation was performed in order to study the chemical composition of the solution in which the AgNPs were synthesized by the here proposed “green” approach, and hence, to elucidate the chemical groups which could be involved in the stabilization of the NPs in aqueous solution upon their formation. For this purpose, the FTIR-ATR spectra of the AgNPs solutions were compared with those of the plant extracts achieved in the same experimental conditions used to synthesize AgNPs. The FTIR-ATR spectrum of AgNPs prepared by the chemical colloidal route, in the presence of sodium citrate and tannic acid surfactant was reported, as a suitable reference for the assignment of the FTIR-ATR peaks of the AgNPs, synthesized by the green route [39].

**Figure 6:**
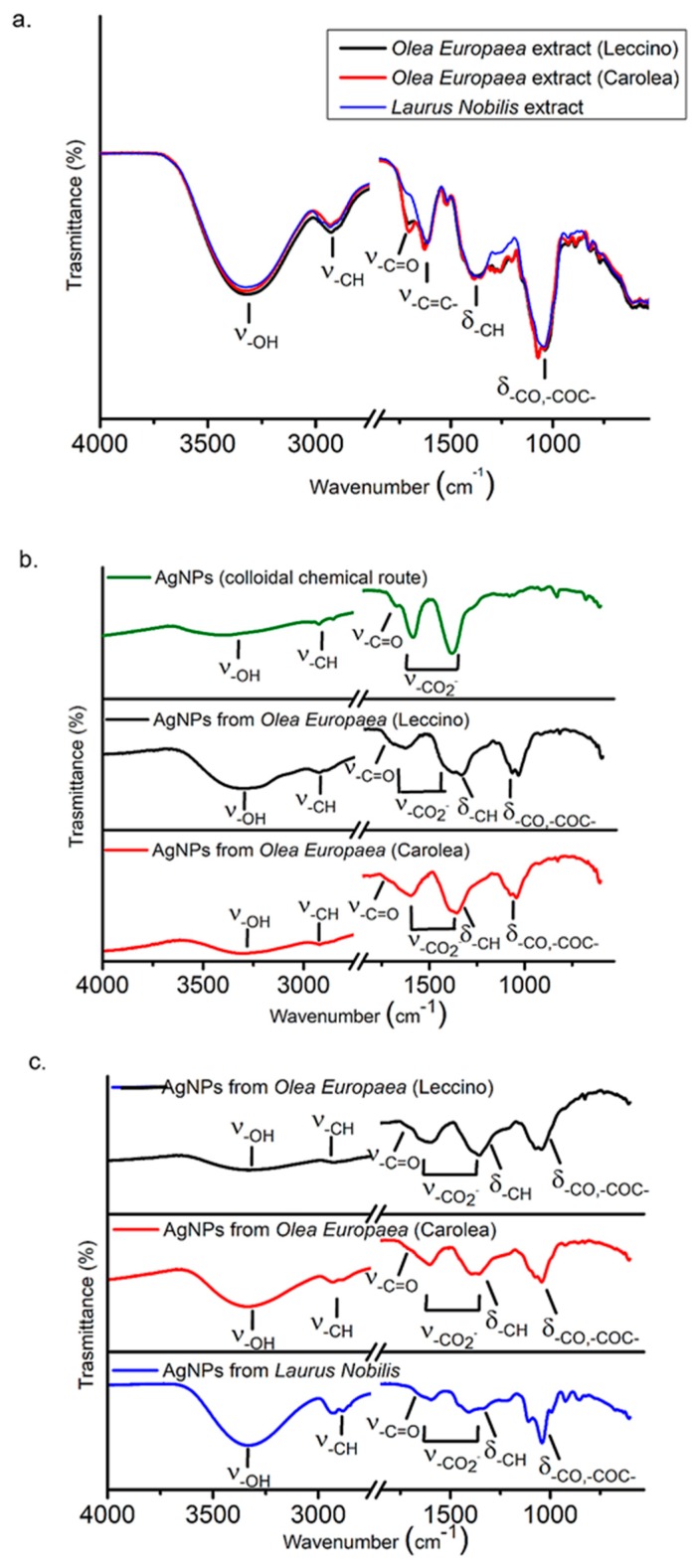
FTIR spectra of *Olea Europaea* (*Leccino* and *Carolea*) and *Laurus Nobilis* extracts (**a**), AgNPs derived from colloidal chemical routes and from green synthesis using *Olea Europaea* extracts (*Leccino* and *Carolea*) at pH 7 (**b**), AgNPs derived from green synthesis using *Olea Europaea* extracts (*Leccino* and *Carolea*) and *Laurus Nobilis* at pH 8 (**c**).

The FTIR-ATR spectra of extracts (Figure 6a) showed, in the high wavenumber region, a broad band in the range of 3700–3014 cm^−1^ and the peaks at 2927 cm^−1^ and 2887 cm^−1^, which were ascribed to the stretching vibrations of O-H and -C-H groups. Such vibrations attested for the presence of molecules containing alcohol, carboxylic acid and aliphatic groups in the plant extracts solutions. On the other hand, in the low wavenumber region, the peaks at ca. 1704 cm^−1^ and 1623 cm^−1^ were ascribed to carboxylic -C=O stretching and aromatic -C=C- ring stretching modes. The peak at 1383 cm^−1^, with the shoulder at 1450 cm^-1^, were aliphatic bending vibrations and the bands at 1261 cm^−1^, 1198 cm^−1^ and 1050 cm^−1^ can be due to the -C-O bending of alcohols and carboxylic acid groups and –C-O-C- stretching vibrations of ethers. The FTIR-ATR spectra of the AgNPs synthetized from *Olea Europaea* (*Leccino* and *Carolea*) at pH 7 (Figure 6b) showed the broad band peak in the range of 3100–3014 cm^−1^ and the peaks at ca. 2927 and 2854 cm^−1^ due to stretching vibrations of the -O-H and aliphatic -C-H groups of the alcohols and carboxylic acids found in the plant extracts solutions (Figure 6a). In the low wavenumber region, they showed a shoulder at 1717 cm^−1^ which can be ascribed to the -COOH stretching of free carboxylic molecules, along with two strong bands, at 1640 cm^−1^ and 1387 cm^−1^ in the NPs from *Leccino*, and 1600 cm^−1^ and 1356 cm^−1^ in the NPs from *Carolea*. Such a double band can be accounted for by the signals of the antisymmetric and symmetric -COO- stretching of carboxylic molecules, respectively, coordinated to the surface Ag atoms [56,57] and thus responsible for the stabilization of the NPs in aqueous solution. Indeed, the same bands were observed also in the FTIR-ATR spectra of AgNPs, synthesized by the colloidal chemical route, by reduction of silver precursor in the presence of sodium citrate and tannic acid surfactants, which were coordinated to the AgNPs surface by their carboxyl groups. Finally, the characteristic peaks of the -C-O bending of alcohols and carboxylic acid groups and –C-O-C stretching vibrations of ethers present in the extracts solutions are still evident in the spectra of the green AgNPs. The AgNPs solutions synthesized at pH 8 (Figure 6c) presented the same vibrations of the NPs solutions achieved at pH 7 (Figure 6b), thus assessing the involvement of molecules containing carboxylic acid groups in the stabilization of the NPs achieved also in these synthesis conditions. The same evidence was obtained for the AgNPs achieved from *Laurus Nobilis* (Figure 6c) in the same experimental conditions, thus indicating that the same chemical moieties were responsible for the stabilization of the AgNPs, irrespectively of the plant from which the synthesis was performed.

The potential toxicity of NPs against cells was then investigated, opting for the MCF-7 and HeLa cell lines. The interaction of AgNPs with MCF-7 was studied because these cells are a well-established model for the identification of adverse effects of NPs and have an epithelial and non-invasive phenotype, as reported elsewhere [58]. In addition, the human cervical carcinoma HeLa cells were selected because they are the most often used models for cytotoxicity studies [59]; in addition this cell line shows good growth and did not require growth factors for its proliferation [60,61].

**Figure 7:**
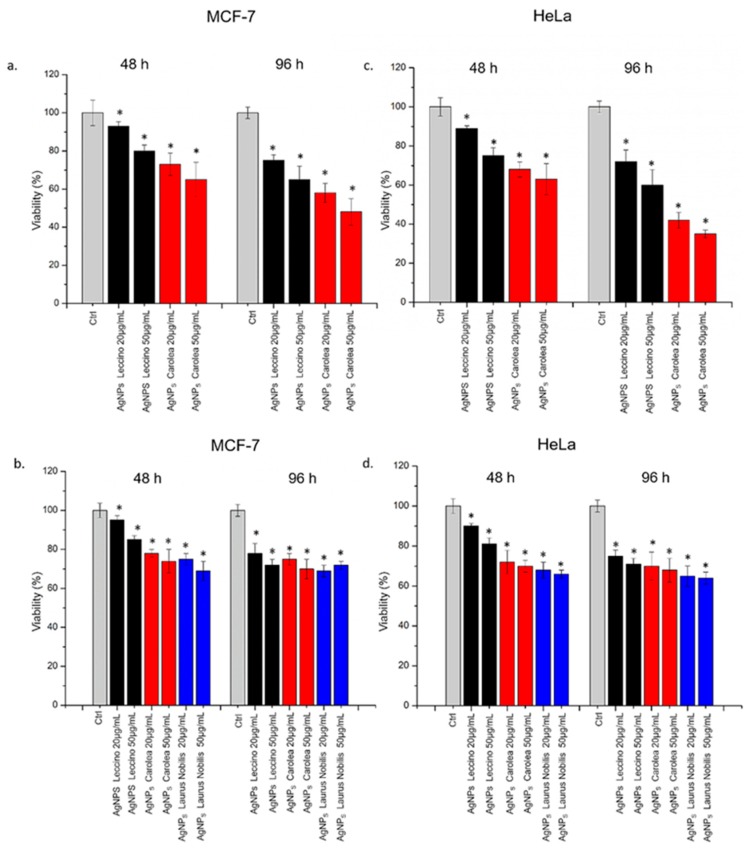
WST-8 of MCF-7 and HeLa cells after 48 h and 96 h exposure to two doses (20 µg/mL and 50 µg/mL) of AgNPs derived from *Oleaea Europeae* (*Leccino* and *Carolea*) at pH 7 (**a** and **c**) and from *Oleaea Europeae* (*Leccino* and *Carolea*) and *Laurus Nobilis* at pH 8 (**b** and **d.**) Viability of NPs-treated cells was normalized to non-treated control cells. As positive control (P), cells were incubated with 5 % Dimethyl Sulfoxide (DMSO) (data not shown). Data reported as mean ± SD from three independent experiments are considered statistically significant compared with control (*n* = 8) for *p* value ˂ 0.05 (< 0.05 *).

We evaluated cell viability after exposing the MCF-7 and HeLa to AgNPs from *Leccino* and *Carolea* at pH 7 (Figure 7a) and to AgNPs from *Leccino*, *Carolea* and *Laurus Nobilis* obtained at pH 8, at concentrations of 20 µg/mL and 50 µg/mL, for 48 h and 96 h (Figure 7b). All the tested NPs induced toxicity in MCF-7 cells, with some differences observed between the AgNPs synthetized at pH 7 and those at pH 8. The cytotoxic effects were more evident when cells were treated with NPs produced at pH 7, especially for *Carolea*-derived NPs, in comparison with the same NPs, obtained at pH 8. In particular, the cells treated with 50 µg/mL of AgNPs prepared from *Carolea* at pH 7 showed a viability reduction of more than 30 % after 48 h, and only 48 % of cells were viable after 96 h. At pH 8, the cytotoxic effects were similar for the AgNPs derived from *Leccino*, *Carolea* and *Laurus Nobilis.*

The treatment against HeLa cells showed the same trend observed for MCF-7 (Figure 7c). Also, in this case, the AgNPs derived from *Carolea* at pH 7 were more toxic than the same obtained from *Leccino*, but the cytotoxic effect was stronger respect to MCF-7. Indeed, at 96 h, AgNPs obtained from *Carolea* induced a viability reduction of 62 % at 50 µg/mL of concentration. Using AgNPs obtained at pH 8, the effects followed the same trend obtained in MCF-7 but the effect resulted more visible in HeLa cells (Figure 7d).

These results suggested a selective toxicity that was dependent on the shape of the NPs, and hence, on the pH used for the synthesis reaction [62,63]. However, it was important to remark that the toxicity induced by these green NPs was lower compared to the same AgNPs obtained from colloidal chemical routes [39].

**Figure 8:**
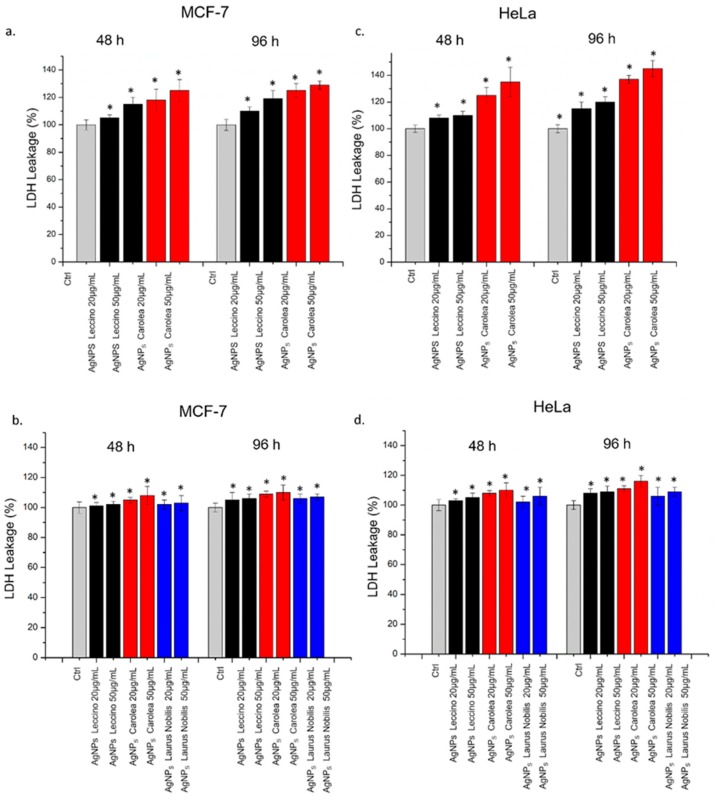
LDH assays on MCF-7 and HeLa cells after 48 h and 96 h exposure to two doses (20 µg/mL and 50 µg/mL) of AgNPs derived from *Oleaea Europeae* (*Leccino* and *Carolea*) at pH 7 (**a** and **c**) and from *Oleaea Europeae* (*Leccino* and *Carolea*) and *Laurus Nobilis* at pH 8 (**b** and **d).** Percent of LDH leakage of NP-treated cells are expressed relative to non-treated control cells. Positive controls (P) consisted in the treatment of cells with 0.9% Triton X-100 showing ca. 500 % LDH increase (data not shown). Data reported as mean ± SD from three independent experiments are considered statistically significant compared with control (*n* = 8) for *p* value ˂ 0.05 (< 0.05 *).

AgNPs induced cell membrane poration with a consequent LDH release in close agreement with the viability results (Figure 8). The effect was more evident in Hela cells with respect to MCF-7 especially upon AgNPs obtained from *Carolea* obtained at pH 7 and after 96 h at the higher concentration (Figure 8a–c). The LDH release percentage reached an increase of about 143 % with respect to the untreated (control) cells after 96 hours of exposure (Figure 8c). Using AgNPs obtained from the three plant extracts at pH 8 (Figure 8b,d), the effects on cell membrane of HeLa and MCF-7 were similar.

**Figure 9:**
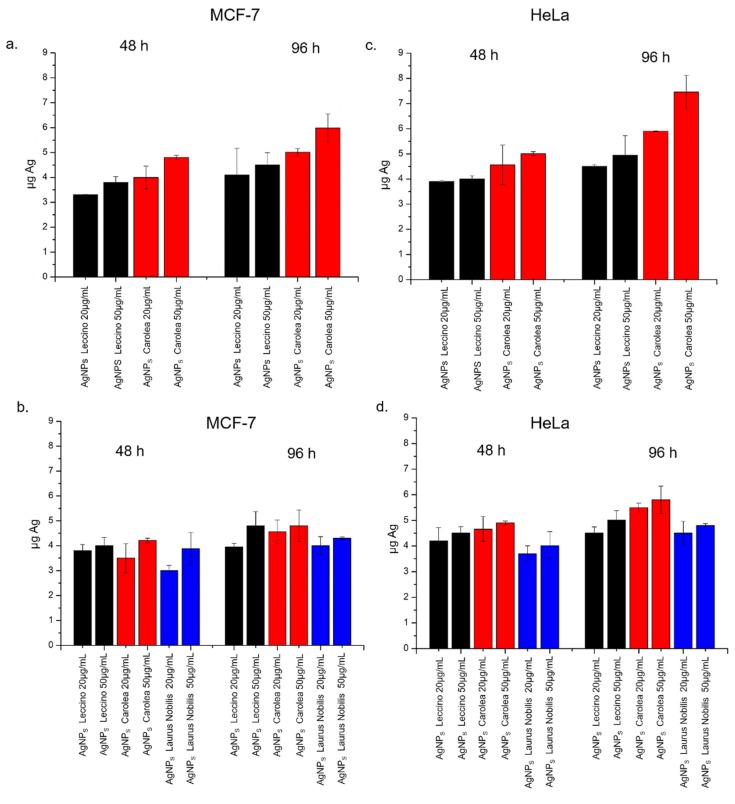
Green AgNPs accumulation in MCF-7 (**a,b**) and HeLa (**c,d**) cell lines exposed to 20 μg/ml and 50 μg/ml of AgNPs derived from *Leccino* and *Carolea* at pH 7 (**a,c**) and AgNPs from *Leccino*, *Carolea*, *Laurus Nobilis* at pH 8 (**b,d**) for 48 h and 96 h. Cells were then harvested, live cells were counted, and Ag content was measured in 360.000 cells (μg Ag). Data reported as mean ± SD from three independent experiments; statistical significance of exposed cells vs. control cells for *p* value < 0.05 (< 0.05 *).

To understand whether the enhanced cytotoxicity of *Carolea*-derived AgNPs may be due to differences in uptake dynamics, we quantified the internalization of green AgNPs (20 µg/mL and 50 µg/mL) in MCF-7 and HeLa by elemental analyses (Figure 9). The uptake was slightly higher for AgNPs derived from *Carolea* compared to those derived from *Leccino* at pH 7 in the two cell lines. AgNPs obtained at pH 8 presented similar uptake levels, which were anyway lower compared to NPs produced at pH 7. In MCF-7, the detected intracellular amount of Ag was (5.93 ± 0.56) µg after incubation with 50 ug/mL of *Carolea*-derived AgNPs for 96 h. In the same conditions of exposure, the Ag content found for the *Leccino*-derived NPs, was of (4.5 ± 0.41) µg (Figure 9a). In HeLa cells, the uptake was more efficient than MCF-7: the effect was more evident for AgNPs from *Carolea* at pH 7: the intracellular Ag measured was (7.4 ± 0.67) µg for cells exposed to 50 ug/mL of NPs for 96 h. The Ag amount observed in HeLa cells exposed to AgNPs obtained from *Leccino* was (4.94 ± 0.78) µg (Figure 9c). The NPs derived from the three extracts at pH 8 shared similar trends of internalization, which were overall lower with respect to the same NPs produced at pH 7 in the two cell lines [64] (Figure 9b,d). The differences in uptake dynamics can be ascribed to the presence of differently shaped NPs, as each shape follows peculiar ways to interact with the cell plasma membrane [47,48]. For example, the high local curvature and irregular shape of *Carolea*-derived AgNPs, having circularity value of (0.55 ± 4), may explain their enhanced cellular internalization rate, compared to spherical NPs having a circularity value near 1. These results could support the idea that AgNPs from *Carolea* cultivar could be a preferable nano-tool for anticancer activity purpose respect to the AgNPs from *Leccino*.

**Figure 10:**
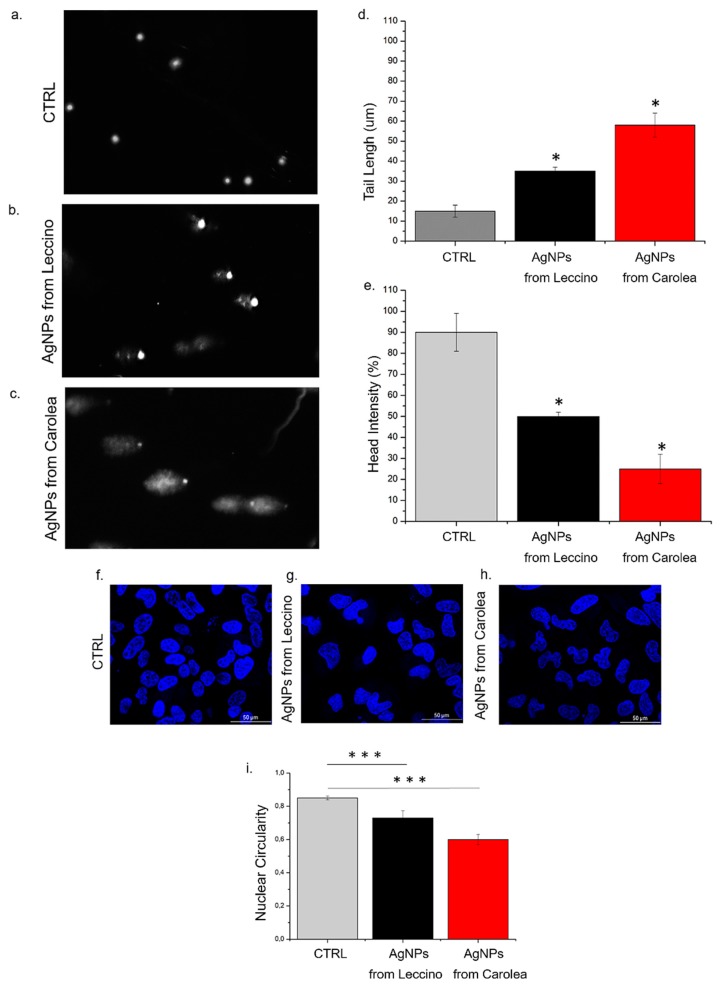
(**a**–**c**). Representative images acquired by Nikon Eclipse Ti fluorescence microscope of AgNPs (derived from *Leccino* and *Carolea*) effect on DNA damage in HeLa cells line. HeLa were treated with NPs (50 µg/mL) for 96 h. DNA damage was evaluated by (**d**) tail length (μm) and (**e**) head intensity (%). Values shown are means from 100 randomly selected comet images of each sample. As a positive control (P) cells were incubated with 500 μM H_2_O_2_ (data not shown). Data are reported as mean ±SD from three independent experiments; *p < 0.05 compared with control (*n* = 3). (**f**–**h**). Representative confocal images of HeLa nuclei: control (**f**) and after the exposure to 50 µg/mL of AgNPs from *Leccino* (**g**) and *Carolea* (**h**) after 96 h. **(i)** Histogram reported the mean values and their respective standard deviation of nuclear circularity. The statistical significance of results respect to control cells was evaluated by *t* test, and reported in histograms (*** *p* < 0.005.).

Once observed that the stronger effect was induced by NPs obtained at pH 7 on HeLa cells, we investigated the effect on DNA using the Comet Assay. As showed in Figure 10a–c the AgNPs from *Leccino* and *Carolea* induced different genotoxicity on HeLa cells using the higher concentration (50 µg/mL) at 96 h. Figure 10b,c clearly showed the differences respect to control (Figure 10a): high level of DNA damage was found after AgNP achieved from *Leccino* and *Carolea* exposure, both in terms of tail length and DNA percentage in the head (Figure 10d,e), showing the typical comet morphology. AgNPs derived from *Carolea* induced a substantial DNA breaks that was evident in the tail length: (59 ± 5) µm after AgNPs-*Carolea* exposure and (35 ± 2) µm for NPs obtained from *Leccino* compared to control (15 ± 3) µm. The greater tail length corresponded to several DNA damage. Contrary, HeLa cells incubated with AgNPs from *Leccino* showed a more evident head percentage (50 ± 2 %) intensity respect to *Carolea* (25 ± 5%) because the DNA was mainly confined in the cellular nuclei. The chromatin remodelling induced an alteration of nuclear morphology that could be quantified in terms of nuclei circularity: in our case, the exposure to AgNPs provoked a circularity value reduction, indicating a pre-apoptotic condition [65]. Confocal acquisitions on Hela cells after the addition of AgNPs (50 µg/mL) up to 96 h showed an alteration of morphology: nuclei become less round and irregular (Figure 10g,h) in comparison with control cells (Figure 10f). Control HeLa cells presented nuclei circularity value of (0.83 ± 0.03). After green AgNPs treatment, a loss of circularity value was observed: the value changed from (0.69 ± 0.06) (AgNPs from *Leccino*) to 0.58 ± 0.05 (AgNPs from *Carolea*).

**Figure 11:**
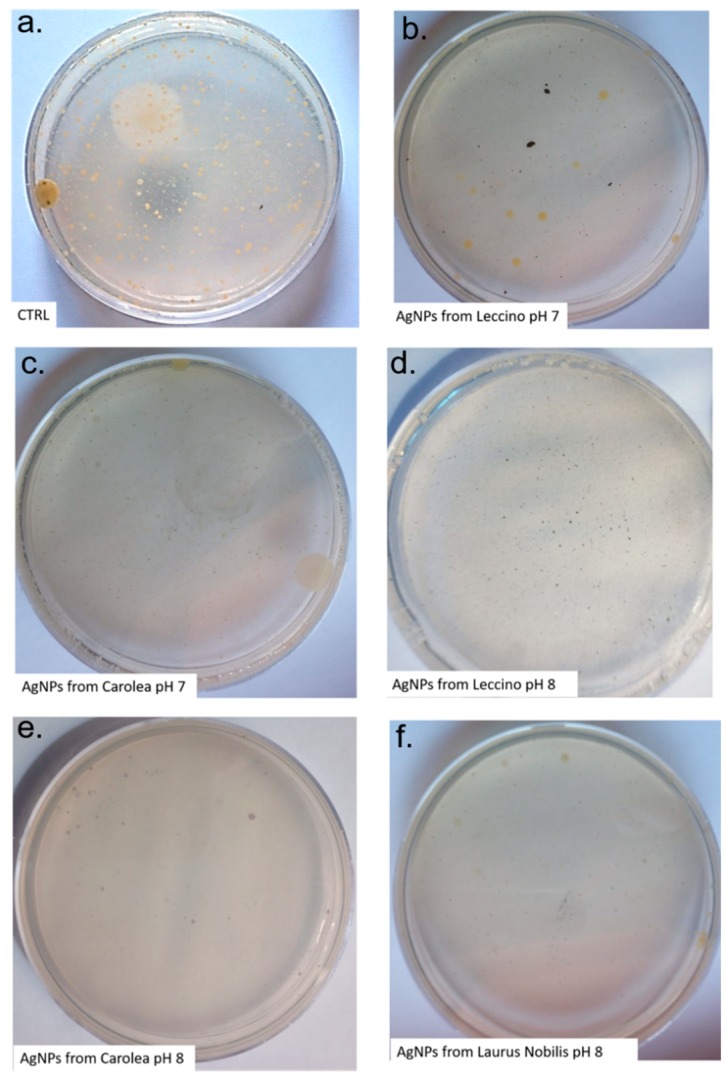
Total bacteria detection by plate count techniques to test colonies at 37 °C. 50 µg/mL of green AgNPs derived from *Leccino*, *Carolea* at pH 7 (**b**,**c**) and from *Leccino*, *Carolea* and *Laurus Nobilis* and pH 8 (**d**,**e**,**f**) were added in water before inoculation to test the antibacterial activity.

We finally tested the antibacterial activities of these NPs. We thus incubated 50 µg/mL of AgNPs in water extracted from a well, containing typical bacteria of well water, e.g., coliforms and faecal coliforms (see the experimental section for details). We tested the total bacterial charge at 22 °C and 37 °C, to assess if the same trend obtained on human cells was maintained. In addition, the anti-coliforms activity was tested. The flora developed at 22 °C is autochthonous in water, while the one that developed at 37 °C can be considered an expression of the presence of bacteria hosted by warm-blooded animals [66]. As reported in EU directives [67], the water designated for human consumption requires absence of microorganisms and the quantitative determination of total colonies and coliforms to exclude the contamination, according to specific guidelines. The total bacteria growth at 37 °C was shown in Figure 11. The count of total bacteria charge at 22 °C and 37 °C, together with total coliforms and faecal coliforms quantification, was reported in Table 3. We observed a pronounced antibacterial activity, which was particularly remarked upon use of the spherical AgNPs, produced at pH 8. The incubation of AgNPs from *Carolea* and *Leccino*, obtained at pH 8, indeed inhibited the growth of bacteria present in the contaminated well water, having high bacterial titer (especially coliforms). These results were in line with those reported in literature [68,69,70], suggesting how the small spherical AgNPs (10–20 nm) showed enhanced antibacterial activity against several bacterial species, compared to the bigger and differently shaped AgNPs. The non-spherical AgNPs produced at pH 7 had lower antibacterial activity with respect to smaller spherical particles. This difference can be explained by the molecular mechanisms guiding the antibacterial effects of AgNPs. This was in fact ascribed to the ability of NPs to release Ag^+^ ions from their surface, a process inducing damages at different level. In particular, they can induce membrane damage and cellular content leakage; in addition, AgNPs or Ag^+^ can bind to the constitutive proteins of cell membrane, involved in transmembrane ATP generation [68]. Hence, the different antibacterial characteristics can be due to potential different rate of Ag^+^ ions release from the particles surface.

**Figure 12:**
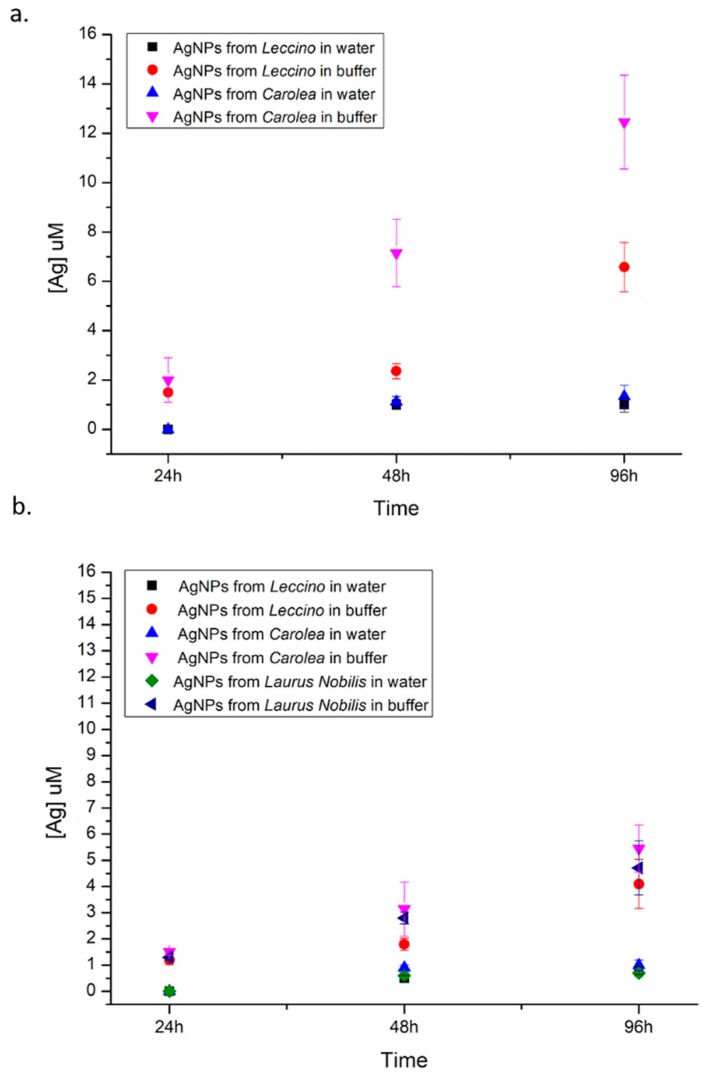
Effects of time and pH on silver ions release from AgNPs (50 μg/ml) derived from *Leccino* and *Carolea* obtained at pH 7 (**a**) and AgNPs from *Leccino*, *Carolea* and *Laurus Nobilis* at pH 8 (**b**). NPs degradation was evaluated both in buffer (pH 4.5) and in water (pH 7) up to 96 h. NPs degradation in neutral conditions was analysed also in culture medium, finding the same behaviour observed in water (data not shown).

In order to understand if the toxicological profile of the green synthetized NPs were associated with their degradation, we have analysed the release of Ag^+^ from AgNPs (50 µg/mL) in water and in acidic buffer that mimic the acidic lysosome environment (pH 4.5). Our results clearly showed a great ions release from *Carolea* obtained at pH 7 in acidic buffer (12.5 ± 1.9) µM after 96 h, whereas in water the release was few both from *Leccino* and *Carolea* AgNPs (Figure 12a): (1 ± 0.3) µM and (1.3± 0.4) µM respectively. The AgNPs obtained at pH 8 showed lower ionization trend even in acidic environment (Figure 12b) with similar results in the three AgNPs species. These results could explain the higher toxicity in cancer cells of AgNPs from *Carolea* obtained at pH 7 after cell internalization, whereas in well water the toxicity against bacteria could be verify due to the interaction of small amount of Ag^+^ released in water. However, this kind of NPs obtained from plants extracts were more resistant to degradation with respect to the NPs obtained with standard chemical route as previously reported [12,39]. Probably the biomolecules adsorbed on NPs surface acted as protection and stabilization agents.

## 4. Conclusions

In this work, a fully green method was reported for the production of AgNPs, completely free from both solvents and hazardous reagents. To do this, leaves extracts from two cultivars of *Olea Europaea* (*Leccino* and *Carolea*) were used, in order to produce AgNPs with a full control of physico-chemical characteristics. In particular, we demonstrated, for the first time, that using either *Leccino* or *Carolea* induces a clear difference in size and shape of AgNPs, in neutral environment; whereas at pH 8, using the same cultivar extracts, the NPs resulted smaller, with more regular morphology and monodispersed. Furthermore, the uptake dynamics and cytotoxicity of these AgNPs were studied in breast cancer cell lines, allowing to prove them as good antibacterial agents, with a further evidence of AgNPs’ different behaviour to induce toxicity in cells and bacteria when obtained at pH 7 or pH 8. Moreover, another strength of this method consists in the theoretically unlimited source of reducing agent (i.e., the leaves extract obtained from agricultural processing waste), as well as its negligible environmental impact.

## Figures and Tables

**Table 1:** ζ-potential values of green silver NPs (AgNPs) achieved at different pH.

Green AgNPs	pH	Zeta Potential Value
AgNPs from *Olea europaea* (*Leccino*)	7	−15 ± 5
AgNPs from *Olea europaea* (*Carolea*)	7	−20 ± 3
AgNPs from *Olea europaea (Leccino*)	8	−30 ± 8
AgNPs from *Olea europaea* (*Carolea*)	8	−18 ± 2
AgNPs from *Laurus Nobilis*	8	−25 ± 2

**Table 2:** Circularity values obtained using ImageJ software on TEM acquisitions.

Green AgNPs	Circularity Value (pH 7)	Circularity Value (pH 8)
AgNPs from *Olea Europaea–(Leccino)*	0.55 ± 4	0.88 ± 3
AgNPs from *Olea Europaea–(Carolea)*	0.28 ± 8	0.63 ± 6
AgNPs from *Laurus Nobilis*	-	0.65 ± 4

**Table 3:** Total colonies at 22 °C and 37 °C as UCF/ml and total coliforms and faecal coliforms as MPN/100ml after exposure to 50 µg/mL of green AgNPs.

Colonies and Coliforms	Contaminated Well Water	Well Water + 50µg/mL AgNPs from Leccino (pH 7)	Well Water + 50µg/mL AgNPs from Carolea (pH 7)	Well Water + 50 µg/mL AgNPs from Leccino (pH 8)	Well Water + 50 µg/mL AgNPs from Carolea (pH 8)	Well Water +50 µg/mL AgNPs from Laurus Nobilis (pH 8)
Total colonies (22 °C)(UCF/ml) 100 allowed	120	108	105	79	82	84
Total colonies (37 °C)(UCF/ml) 20 allowed	28	12	6	absent	1	3
Total Coliforms (MPN/100 mL) Absent allowed	19	11	9	absent	absent	absent
Total Faecal Coliforms (MPN/100 mL) Absent allowed	7	6	6	absent	absent	absent
